# Understanding of tolerance in TRAIL-induced apoptosis and cancelation of its machinery by α-mangostin, a xanthone derivative

**DOI:** 10.18632/oncotarget.4558

**Published:** 2015-07-16

**Authors:** Minami Kumazaki, Haruka Shinohara, Kohei Taniguchi, Hiroshi Ueda, Mayuko Nishi, Akihide Ryo, Yukihiro Akao

**Affiliations:** ^1^ United Graduate School of Drug Discovery and Medical Information Sciences, Gifu University, Yanagido Gifu 501-1193, Japan; ^2^ Department of Microbiology, Yokohama City University School of Medicine, Yokohama 236-0027, Japan

**Keywords:** TRAIL-induced apoptosis, TRAIL-resistance, α-mangostin, miR-133b, cancer stem-like cell

## Abstract

Tumor necrosis-factor (TNF)-related apoptosis-inducing ligand (TRAIL) is a member of the TNF-superfamily that selectively induces apoptosis through death receptors (DRs) 4 and/or 5 in cancer cells. These receptors are expressed on the cancer cell surface, without affecting normal cells. Unfortunately, many clinical studies have shown that cancer cells acquire TRAIL-resistance and finally avoid TRAIL-induced apoptosis. The detailed mechanisms of this resistance are not well understood. In the current study, we established a TRAIL-resistant human colon cancer DLD-1 cell line to clarify the mechanisms of TRAIL-resistance and developed agents to cancel its machinery. Also, we found that cancer stem-like cells from breast epithelial proliferating MCF10A cells were also sensitive to TRAIL-induced apoptosis. The enforced expression of DR5 in both TRAIL-resistant cells partially recovered the sensitivity to the TRAIL ligand, which was judged by the activation of caspase-8. As a result, we newly found that the mechanisms of TRAIL-resistance comprised co-existence of a decrease in the expression level of DR5 along with malfunction of its recruitment to the cell surface, as evidenced by Western blot and immunocytological analysis, respectively. Interestingly, α-mangostin, which is a xanthone derivative, canceled the resistance by increasing the expression level of DR5 through down-regulation of miR-133b and effectively induced the translocation of DR5 to the cancer cell surface membrane in TRAIL-resistant DLD-1 cells. These findings indicate that α-mangostin functioned as a sensitizer of TRAIL-induced apoptosis and may thus serve as a possible adjuvant compound for cytokine therapy to conquer TRAIL-resistance.

## INTRODUCTION

After its discovery in 1995, tumor necrosis factor (TNF)-related apoptosis-inducing ligand (TRAIL/Apo2L) was identified to be a member of the TNF superfamily [1]. TRAIL is a type 2 trans-membrane protein that functions in extracellular signaling by acting through death receptors DR4 and/or DR5 [2]. Upon being stimulated by this ligand, DRs recruit Fas-associated death domain (FADD) protein and the initiator caspase-8, resulting in the formation of the death-inducing signaling complex (DISC). The recruited capase-8 undergoes autocatalytic cleavage and activation to trigger the cascade that ultimately leads to apoptosis [3]. With virtually no toxicity toward normal cells, recombinant human TRAIL or agonistic antibodies specifically targeting DR5 are currently being tested in several clinical trials. Unfortunately, their application for anticancer treatment is limited because of resistance to the development to TRAIL-induced apoptosis and their short half-life in serum [4]. The reason why cancer cells are resistant to TRAIL-induced apoptosis is not yet known, but many clinical studies have attributed TRAIL-resistance to the down-regulation of TRAIL receptors [5] and to mutation of caspase-8 [6]. Recently, clinical trials of combination treatment with anti-DR5 antibody and chemotherapeutics have been under investigation; however, such an approach seems hopeless because of hepatic dysfunction and adverse effects [7]. So far, the use of phytochemicals as anti-cancer agents has gained high importance for chemoprevention and treatment, as indicated by many studies [8]. We also reported that naturally occurring chemo-preventive compounds such as resveratrol and α-mangostin exhibit growth inhibition by inducing apoptosis through a dual effect, i.e., the modulation of intracellular signaling transduction pathways involved in both apoptosis and proliferation [9]. α-Mangostin is a xanthone isolated from the pericarps of the mangosteen fruit. It has been used as a traditional medicine for treatment of skin infections and wounds in Southeast Asia for many years. In this study, we comprehensively examined the mechanisms underlying TRAIL-resistance by using TRAIL-sensitive and -resistant cell lines and developed natural compounds to cancel this resistance. On the other hand, we also tried to clarify whether cancer stem cells would be sensitive to TRAIL-apoptosis and show increased sensitivity in the presence of α-mangostin. As a result, we newly found that the mechanism of TRAIL-resistance consisted of a decrease in the expression level of DR5 and malfunction of its recruitment to the cell surface. Interestingly, α-mangostin canceled the resistance by increasing the expression level of DR5 through down-regulation of miR-133b and effectively induced the translocation of DR5 to the cell surface in DLD-1 cells. Also, α-mangostin enhanced the TRAIL-apoptosis in cancer stem-like cells from MCF10A. These findings altogether indicate that α-mangostin functioned as a sensitizer of TRAIL-induced apoptosis and may be used as a possible adjuvant compound for cytokine therapy to conquer TRAIL-resistance.

## RESULTS

### Mechanism of TRAIL-resistance in human colon cancer cells

**Figure 1:**
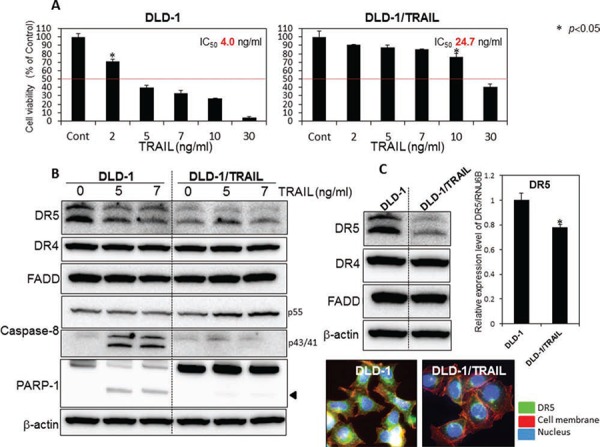
The mechanism of TRAIL-resistance was due to down-regulation of DR5 and malfunction of its recruitment **A.** TRAIL-sensitive DLD-1 and -resistant DLD-1 cells were treated with rTRAIL (2, 5, 7, 10, 30 ng/ml) for 24 h. The cell viability was estimated at 24 h after the treatment. Data were obtained from 3 independent experiments. The cell viability of the control (0; DMSO alone) is indicated as 100%. The growth inhibitory activity (IC_50_) of each compound is indicated in each panel. **B.** Western blot analysis was performed to determine the expression levels of DR5, DR4, FADD, caspase-8, and PARP-1 after the treatment with rTRAIL (5 and 7 ng/ml), with β-actin used as an internal control. **C.** Western blot analysis was performed to determine steady-state expression of DR5, DR4, and adaptor molecule FADD. β-actin was used as an internal control. Also shown are the steady-state expression levels of DR5 mRNA as relative ratios with respect to the GAPDH expression level. The expression level of mRNA was calculated by the ΔΔCt method. Means (S.D. indicated by error bars are shown. Lower photomicrographs: The photomicrograph shows the results of immunofluorescence staining for DR5 (anti-DR5) on the cell surface and in the cytosol of DLD-1 and DLD-1/TRAIL cells. Nuclei were counterstained in blue with Hoechst33342.

At the start of this study, we established a TRAIL-resistant human colon cancer DLD-1 cell line (DLD-1/TRAIL). We examined the anti-proliferative effect of rTRAIL at various concentrations on human colon cancer DLD-1 and DLD-1/TRAIL cells as judged from the cell viability assessed by use of the trypan blue dye-exclusion test. The IC_50_ value of rTRAIL for DLD-1/TRAIL was approximately 25 ng/ml, which was 6 times higher than that for the parental DLD-1 cells (Fig. 1A). Firstly, we sought to elucidate the mechanism of this TRAIL-resistance by using both cell lines. As shown in Fig. 1B, the activation of caspase-8 and appearance of the cleaved form of PARP-1 were clearly observed at 48 h after the treatment of the TRAIL-sensitive cells with rTRAIL. However, this was not the case for the DLD-1/TRAIL cells. Many clinical studies have attributed TRAIL-resistance to the down-regulation of TRAIL receptors. Therefore, we evaluated the steady-state expression levels of DR5, DR4, procaspase-8 and adaptor molecule FADD by performing Western blot analysis (Fig. 1C). DR5 was markedly down-regulated in DLD-1/TRAIL cells compared with its expression in DLD-1 cells. On the other hand, the expression levels of DR4 and FADD were almost unchanged. Also, we confirmed that the apoptosis induced by rTRAIL was due to the extrinsic pathway via caspase-8 activation. Next, we examined the oligomerization of DR5 on the cell surface from the cytoplasm by performing immunofluorescence staining (Fig. 1C Lower). As shown in this figure, the intensity of immunofluorescence emitted from the cell surface, indicating oligomerized DR5, was markedly decreased in the TRAIL-resistant cells compared with that for TRAIL-sensitive DLD-1 cells. Thus, the impaired oligomerization of DR5 was also involved in the mechanism of TRAIL-resistance. These results altogether indicated that the mechanism of TRAIL-resistant was due to the down-regulation of DR5 and the impaired oligomerization of DR5 on the cell surface.

### Growth inhibition by combined treatment with α-mangostin and rTRAIL

**Figure 2:**
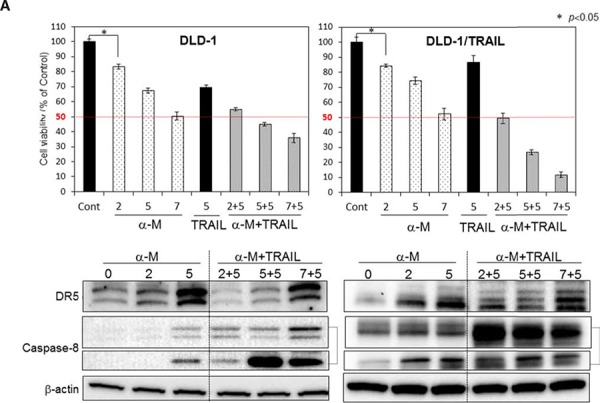
α-Mangostin and rTRAIL significantly induced growth suppression by causing up-regulation of DR5 TRAIL-sensitive and -resistant DLD-1 cells were treated with α-mangostin (2, 5, 7 μM) and/or rTRAIL (5 ng/ml) for 48 h. The cell viability was estimated at 48 h after the treatment. Data were obtained from 3 independent experiments. The cell viability of the control (0; DMSO alone) is indicated as 100%. Western blot analysis was performed to determine the level of DR5 and activation of caspase-8. β-actin was used as an internal control.

Recently, a few clinical trials using combination treatment with anti-DR5 antibody and chemotherapeutics were undertaken; however, the results have been discouraging because of hepatic dysfunction and other adverse effects [7]. Much evidence has indicated the use of phytochemicals to be beneficial for cancer prevention. To explore the use of such compounds as a possible adjuvant for cytokine therapy to conquer TRAIL-resistance, we examined the anti-proliferative effect of 5 representative phytochemicals, as judged form their effect on cell viability assessed by use of the trypan blue dye-exclusion test (Supplementary Fig. 1). Among the compounds tested, α-mangostin enhanced TRAIL-induced apoptosis the greatest. Therefore, we focused on α-mangostin, a xanthone derivative, and exhibited its effect on various types of cancer cells. First, we examined the synergistic anti-proliferative effects of α-mangostin and rTRAIL on DLD-1 and DLD-1/TRAIL cells by incubating them for 48 h with various concentrations of α-mangostin (0, 2, 5, 7 μM) and/or rTRAIL (5 ng/ml) (Fig. 2). The growth was significantly suppressed in a concentration-dependent manner by the treatment with α-mangostin. The IC_50_ value of α-mangostin for DLD-1/TRAIL cells was approximate 7 μM, which was similar to that in the parent TRAIL-sensitive DLD-1 cell line. For the combination treatment with α-mangostin and rTRAIL, the concentration of rTRAIL was fixed at 5 ng/ml, which did not exhibit the growth suppression in DLD-1/TRAIL cells. As a result, the combination treatment with α-mangostin and rTRAIL resulted in synergistic growth suppression in DLD-1/TRAIL cells. Also, α-mangostin dramatically up-regulated the expression level of DR5 in the TRAIL-resistant cell line. These results indicated that α-mangostin elevated TRAIL-sensitivity by up-regulating the expression level of DR5.

### α-Mangostin effectively induced DR5 oligomerization on the cell surface membrane

**Figure 3:**
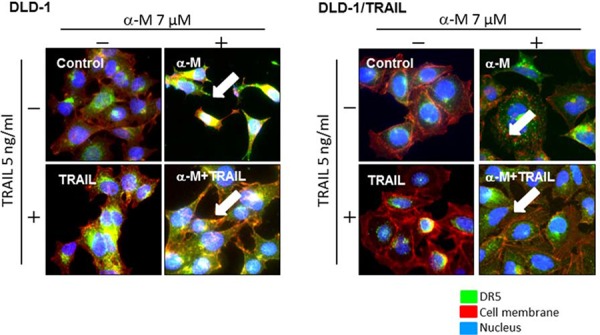
α-Mangostin effectively induced DR5 oligomerization at the cell surface membrane TRAIL-sensitive and -resistant DLD-1 cells were treated with α-mangostin (7 μM) and/or rTRAIL (5 ng/ml) for 48 h. The results of immunofluorescence staining for anti-DR5 binding on the cell surface and in the cytosol of untreated (Control; DMSO) or α-mangostin and /or rTRAIL treated cells are shown. Nuclei were counterstained in blue with Hoechst33342. Anti-DR5 bound to the cell surface is indicated by white arrows.

It has been reported that high levels of basal autophagosomes and DR5 co-localized with LC3-II in these autophagosomes are the reason why the expression of DR5 is down-regulated, which explanation has been validated in TRAIL-resistant breast cancer cell lines [10]. In the case of DLD-1/TRAIL cells, the transition from LC3-I to LC3-II was not observed (Supplementary Fig. 2). So far, DR5 receptors were found to be mislocalized in intracellular compartments that are yet to be characterized. To visualize intracellular and surface binding of anti-DR5 antibody in untreated or α-mangostin (7 μM)- and/or rTRAIL (5 ng/ml)-treated cells, we performed immunofluorescence staining (Fig. 3) and observed increased binding of DR5 to the cell surface membrane of cells treated with α-mangostin alone and to that of those incubated with the combination of α-mangostin and rTRAIL. These results were not observed when TRAIL alone was used. As shown in the figure, the efficiency of oligomerization in DLD-1/TRAIL cell was slightly lower than that in the TRAIL-sensitive ones. These results indicated that α-mangostin effectively induced the translocation from the cytoplasm to the surface membrane of the tumour cells.

### Up-regulation of DR5 by α-mangostin contributed to the enhancement of TRAIL-induced apoptosis

**Figure 4:**
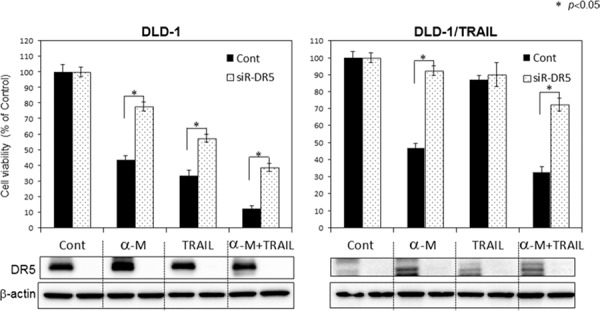
Gene silencing of DR5 suppressed growth inhibition induced by combined treatment with α-mangostin and rTRAIL **A.** TRAIL-sensitive and -resistant DLD-1 cells were transfected with control or DR5 siRNA (2 nM) plasmids for 48 h and then treated with α-mangostin (7 μM) and/or rTRAIL (5 ng/ml) for 48 h. Western blot analysis was performed to determine the expression of DR5, and β-actin was used as an internal control.

In recent years, numerous chemotherapeutics, natural products and newly synthesized molecules have been screened for their ability to restore TRAIL sensitivity in cancer cells. Among natural products, polyphenols and a particular subgroup of flavonoids constitute the major category of molecules used in combination with TRAIL for the treatment of human colon cancer cells. Food polyphenols sensitize human colon cancer cells to TRAIL-driven cell death mainly by increasing the expression of DR5 and to a lesser extent that of DR4 [11]. To further confirm the role of α-mangostin in DR5 up-regulation, we performed gene silencing of DR5 by using its siRNA. DLD-1 cells and DLD-1/TRAIL cells transfected with the control miRNA or siRNA for DR5 (siR-DR5) were co-treated with α-mangostin (7 μM) and/or rTRAIL (5 ng/ml) for 48 h (Fig. 4). As shown in the figure, the transfection of DLD-1 or DLD-1/TRAIL cells with siR-DR5 resulted in a complete suppression of DR5 expression even in the case when the cells had been pre-treated with α-mangostin compared with the expression in the cells transfected with the control miRNA. Control miRNA-transfected cells showed about 60% growth suppression following the treatment with α-mangostin; however, transfection of the DLD-1/TRAIL cells with siR-DR5 decreased this suppression to approximately 10%. In all cases, treatment with siR-DR5 resulted in the higher cell viability, and the difference in viability between the control miRNA and siR-DR5 was significant in the α-mangostin plus rTRAIL group. These results indicated that the up-regulation of DR5 by α-mangostin contributed to the enhancement of TRAIL-induced apoptosis in both DLD-1 and DLD-1/TRAIL cells.

### Up-regulation of DR5 by α-mangostin was due to down-regulation of miR-133b

**Figure 5:**
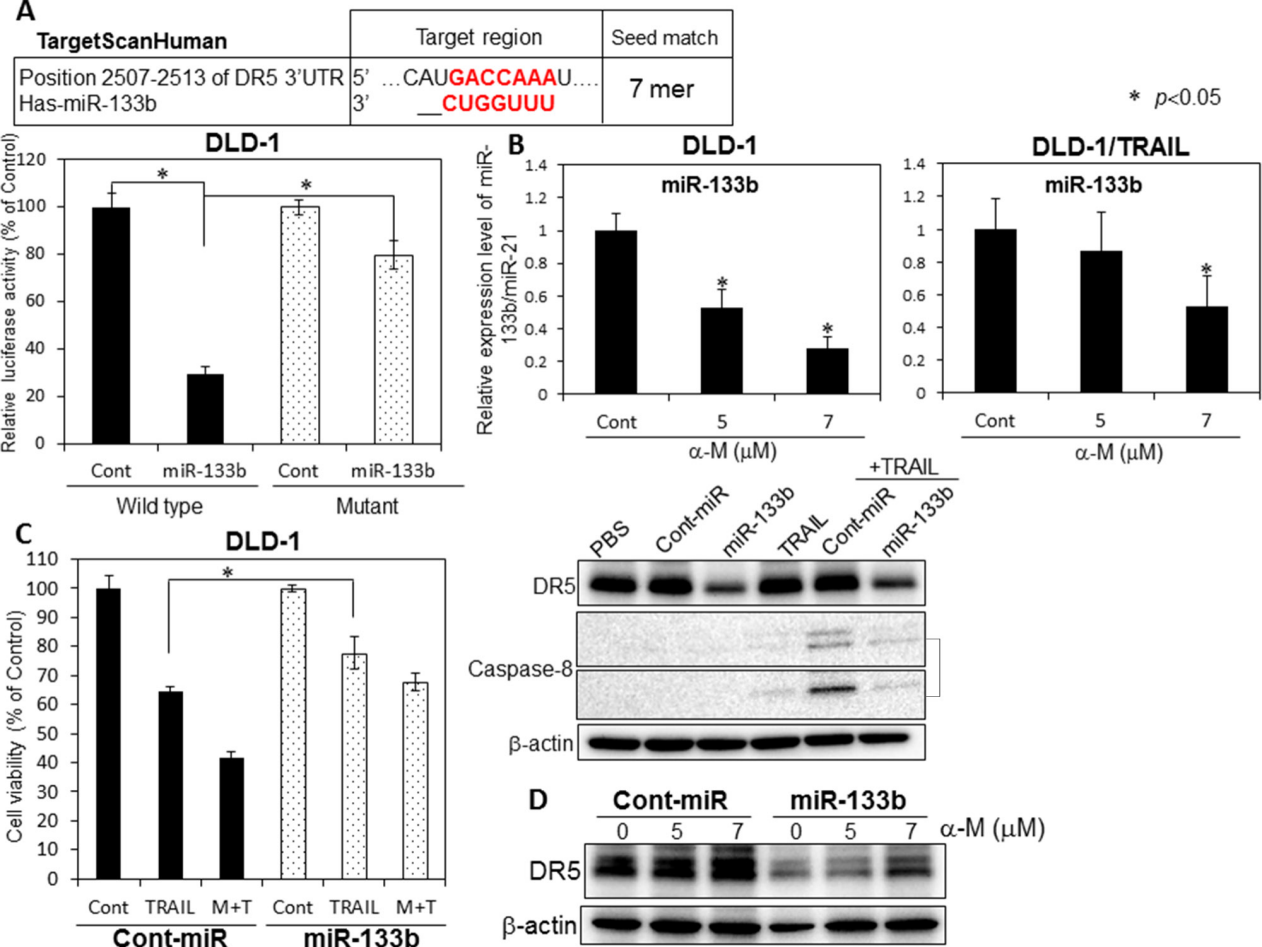
α-Mangostin cased a decrease in the expression level of miR-133b, which targets DR5 **A.** The vector with the binding site for miR-133b is indicated as Wild (+) type; and that without it, as Mutant. **B.** Expression level of miR-133b in TRAIL-sensitive and -resistant DLD-1 cells treated with α-mangostin (5, 7 μM), as evaluated by RT-qPCR. The expression levels were calculated by the ΔΔ*Ct* method. **C.** DLD-1 cells were transfected with control or miR-133b (10 nM) for 48 h, and then exposed to α-mangostin (7 μM) and/or rTRAIL (5 ng/ml) for 24 h. The cell viability was estimated at 48 h after the treatment. Data were obtained from 3 independent experiments. The cell viability of the control (0; DMSO alone) is indicated as 100%. Western blot analysis was performed to determine the expression of DR5 and the active form of caspase-8, with β-actin use as an internal control. **D.** Control and miR-133b (10 nM) were transfected into DLD-1 cells for 48 h, and the cells were then exposed to α-mangostin (5, 7 μM). Western blot analysis was performed to determine the expression of DR5. β-actin was used as an internal control.

So far, we previously reported that miR-143 is an anti-oncogenic miRNA and that its expression level is increased by α-mangostin [12]. Also, over 100 miRNAs, involved in the control of different cellular processes such as inflammation and apoptosis, were shown to be as modulated by polyphenols [13]. According to the *in-silico* prediction tools TargetScan, DR5 (TNFRSF10B) displays a single miR-133b binding site in its 3′-UTR. More recently, protumorigenic role of miR-133b was evidenced in cervical cancer: miR-133b directly regulated anti-apoptotic gene Fas apoptosis inhibitory molecule (FAIM) [14]. In order to validate the target gene of miR-133b as being DR5, we performed a luciferase reporter assay (Fig. 5A). The co-transfection with miR-133b and the pMIR sensor vector, which included the candidate target region bound by miR-133b, resulted in significant inhibition of the luciferase activity compared with the co-transfection with control miRNA, but not in the case of the pMIR sensor vector that include the region without the binding site. Furthermore, mutations of the DR5 3′-UTR binding site significantly abolished the ability of miR-133b to decrease the luciferase activity. The results of this assay demonstrated that miR-133b targets DR5. When we examined the intracellular level of miR-133b at 48 h after the treatment with α-mangostin (Fig. 5B), we found that α-mangostin significantly down-regulated the level of miR-133b in both TRAIL-sensitive DLD-1 and DLD-1/TRAIL cell lines. We further examined whether miR-133b was associated with TRAIL-induced apoptosis. DLD-1 cells transfected with the control miRNA or miR-133b were treated with α-mangostin (7 μM) and/or rTRAIL (5 ng/ml) for 48 h. As shown in the Fig. 5C, transfection of the cells with miR-133b resulted in a significant cancellation of the growth suppression induced by the combination of α-mangostin and rTRAIL. Furthermore, the activation of caspase-8 was impaired in the miR-133b-transfected cells. Furthermore, we examined the cancelling effect of miR-133b on the up-regulated expression of DR5 after the treatment with α-mangostin to validate the relationship between the up-regulation of DR5 by α-mangostin and the down-regulation by miR-133b. As shown in Fig. 5D, 10 nM miR-133b clearly reversed the up-regulation of DR5 induced by α-mangostin. All of these results taken indicated that α-mangostin cancelled the resistance to TRAIL by increasing the expression level of DR5 through down-regulation of miR-133b.

### Mechanism of TRAIL-resistance in human mammary gland epithelial cells

**Figure 6:**
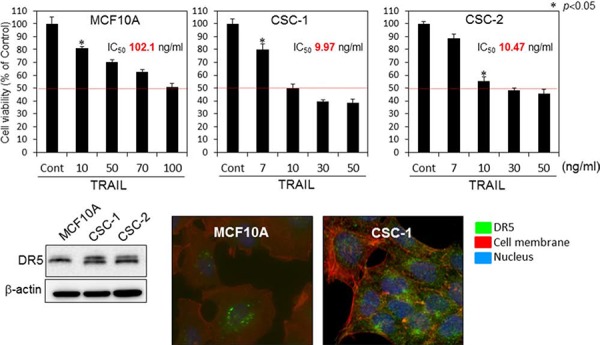
TRAIL-resistance of human mammary gland epithelial cells was due to malfunction of its recruitment to the cell surface membrane Top graphs; Breast cancer stem-like CSC-1 and the parent MCF10A cells were treated with rTRAIL (7, 10, 30, 50, 70, 100 ng/ml) for 24 h. The cell viability was estimated at 24 h after the treatment. Data were obtained from 3 independent experiments. The cell viability of the control (0; DMSO alone) is indicated as 100%. The growth inhibitory activity (IC_50_) of each compound is indicated in each panel. Lower blot: Western blot analysis was performed to determine the steady-state expression of DR5, β-actin as the internal control. Lower photomicrographs: Oligomerization of DR5. Nuclei were counterstained in blue with Hoechst33342. Anti-DR5 bound to the cell surface is indicated by white arrows

In order to further validate the mechanism of TRAIL-resistance in another cell line and to examine the sensitivity to TRAIL-induced apoptosis in breast cancer stem-like cells (CSC-1 and -2), we performed similar experiments by using these cells and their parent non-cancerous human mammary gland epitherial (MCF10A) cells [15]. The anti-proliferative effect of rTRAIL at various concentrations on both paired cell lines are shown in Fig. 6. The IC_50_ value of rTRAIL for MCF10A cells was approximately 100 nM, which was 10 times higher than that for the CSC-1 and -2 cells. We evaluated the steady-state expression levels of DR5, DR4, and adaptor molecule FADD by performing Western blot analysis (Fig. 6 Lower blot). Similar expression profiles of DR5 were found for both CSC-1 and -2 cell lines. The expression levels of DR4 and FADD were almost unchanged. With regards to the MCF10A cells, the upper band, which would be the DR5 precursor, was faint. Thus, these 2 cancer stem-like cell lines were sensitive to TRAIL-induced apoptosis. Next, we examined the oligomerization of DR5 on the cell surface by performing immunofluorescence staining after rTRAIL treatment of MCF10A and CSC-1 cells (Fig. 6 Lower photomicrographs). As shown in this figure, the intensity of fluorescence indicating DR5 on cell the surface was markedly less in the MCF10A cell line. These results altogether indicated that the main mechanism of TRAIL-resistant was the malfunction of DR5 recruitment to the cell surface. Also, cancer stem cells would be induced to undergo apoptosis by the TRAIL/DR5/caspase-8 signaling pathway.

### Growth inhibition of cancer stem-like CSC cells by combined treatment with α-mangostin and rTRAIL

**Figure 7:**
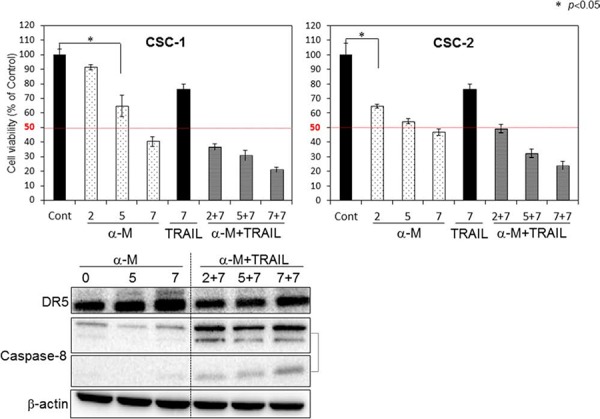
α-Mangostin and rTRAIL induced significant growth suppression of CSC-1 and -2 cells by up-regulating expression of DR5 Upper bar graphs: Breast cancer stem-like CSC-1 and CSC-2 cells were treated with α-mangostin (2, 5, 7 μM) and/or rTRAIL (7 ng/ml) for 48 h. The cell viability was estimated at 48 h after the treatment. Data were obtained from 3 independent experiments. The cell viability of the control (0; DMSO alone) is indicated as 100%. Lower blot: Western blot analysis was performed to determine the expression of DR5 and activation of caspase-8, with β-actin as the internal control.

**Figure 8:**
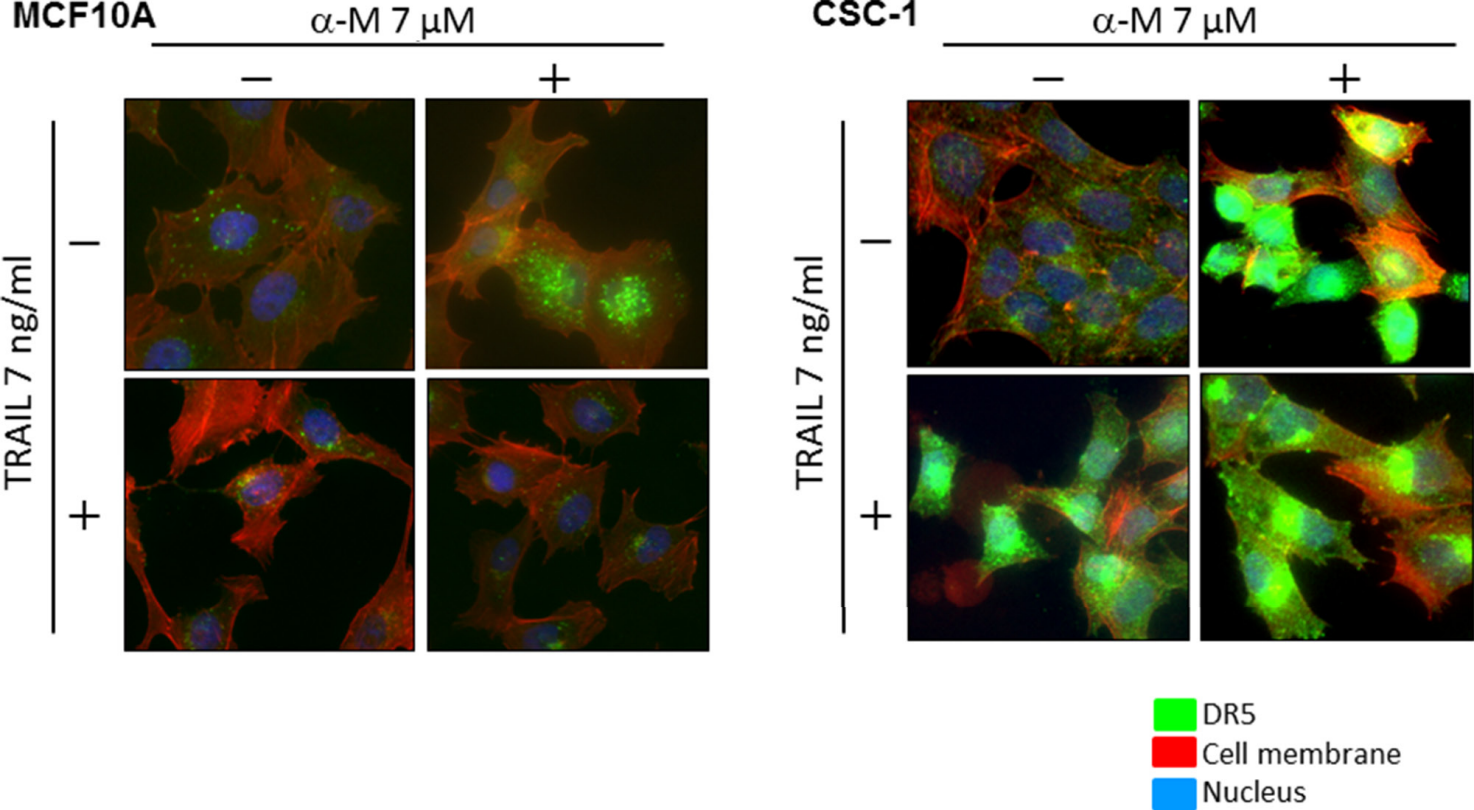
α-Mangostin effectively induced DR5 oligomerization at the cell surface membrane of cancer stem-like cell Breast cancer stem like CSC-1 and the parent MCF10A cells were treated with α-mangostin (7 μM) and/or rTRAIL (7 ng/ml) for 48 h. The results of immunofluorescence staining for DR5 bound on the cell surface and in the cytosol of untreated (Control; DMSO alone) or α-mangostin- and/or rTRAIL-treated cells. Nuclei were counterstained in blue with Hoechst33342.

To explore a possible adjuvant compound for cytokine therapy to induce sensitization to TRAIL-induced apoptosis in CSC cells, we examined the synergistic anti-proliferative effects of α-mangostin and rTRAIL by treating the cells with various concentrations of α-mangostin (0, 2, 5, 7 μM), and/or TRAIL 7 ng/ml for 48 h (Fig. 7). Their growth was significantly suppressed in a concentration-dependent manner by the treatment with α-mangostin. The IC_50_ value of α-mangostin for CSC-1 cells was approximately 6 μM, which was similar to that for the DLD-1 cell line. Upon the combination treatment with α-mangostin and rTRAIL, the concentration of rTRAIL was fixed at 7 ng/ml, which did not exhibit the growth suppression of CSC-1 cells. As a result, the combination treatment with α-mangostin and rTRAIL resulted in synergistic growth suppression in these cells. Also, the expression level of DR5 was up-regulated by α-mangostin, and the activation of caspase-8 was increased, by combination treatment with α-mangostin and rTRAIL. Furthermore, α-mangostin increased the binding of DR5 to the cell surface membrane of the CSC-1 cells treated with α-mangostin alone or with the combination of α-mangostin and rTRAIL. These results altogether indicated that α-mangostin functioned as a sensitizer of TRAIL-induced apoptosis by up-regulating the expression level of DR5 in, and affording efficient translocation of DR5 from the cytoplasm to the tumor cell surface membrane of, CSC-1 cells (Fig. 8)

### Induction of TRAIL-sensitivity by overexpression of DR5 in TRAIL-resistant DLD-1/TRAIL and MCF10A cells

**Figure 9:**
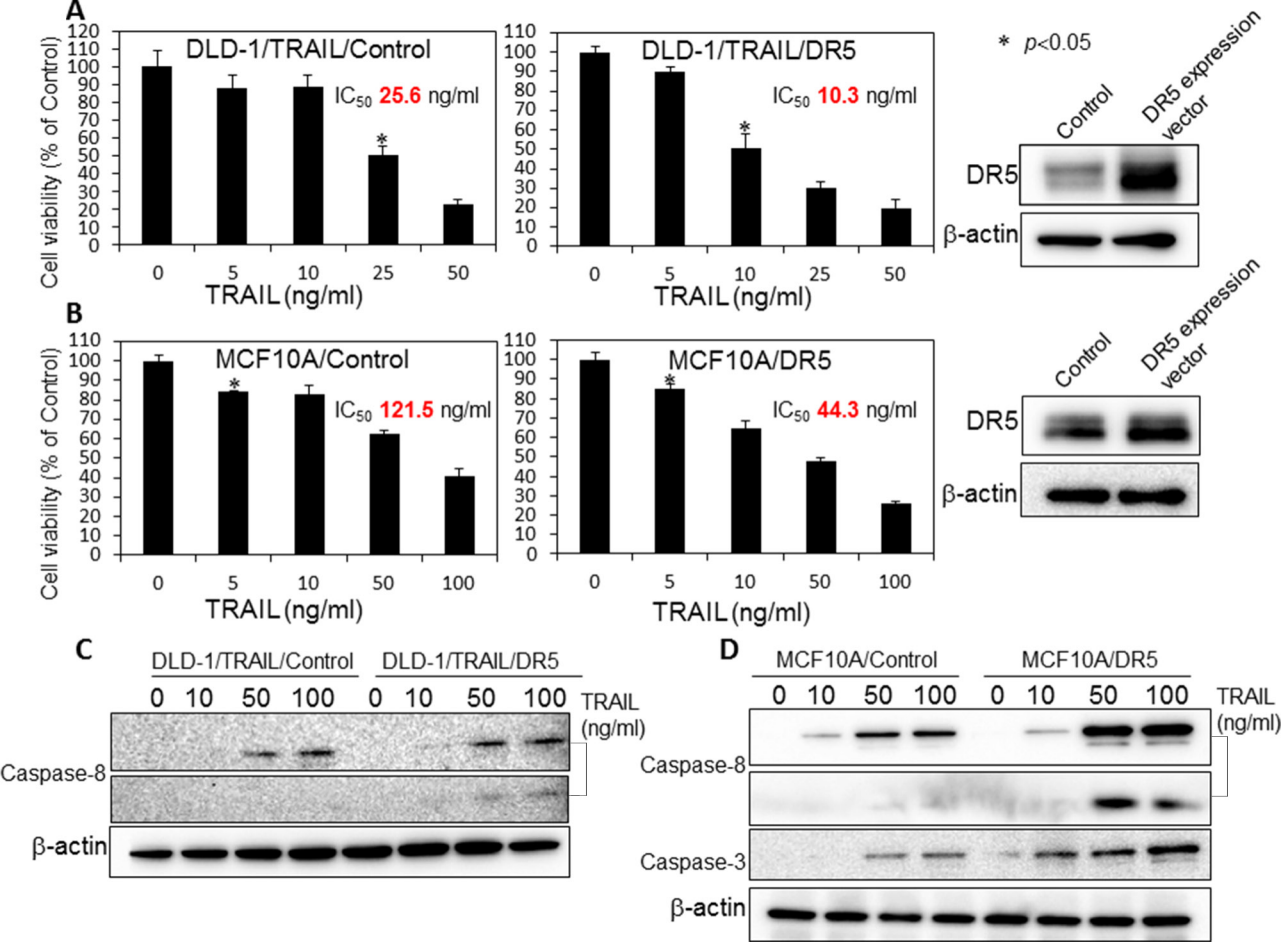
Overexpression of DR5 induced TRAIL sensitivity in TRAIL-resistant DLD-1/TRAIL and MCF10A cells **A.** Control and DR5 expression plasmid vectors (0.4 μg/ml) were used to transfect DLD-1/TRAIL cells for 24 h, and the cells were then exposed to rTRAIL (5, 10, 25, 50 ng/ml) for 24 h. **B.** MCF10A cells were transfected with control or DR5 expression plasmid vectors (0.4 μg/ml) for 24 h, and then exposed to rTRAIL (5, 10, 50, 100 ng/ml) for 24 h. The cell viability was estimated at 24 h after the treatment. Data were obtained from 3 independent experiments. The cell viability of the control (0; PBS alone) is indicated as 100%. **C** and **D.** Western blot analysis was performed to determine the expression of DR5, activation of caspase-8 and caspase-3 with β-actin used as the internal control.

In order to confirm that the decreased expression of DR5 was one of mechanisms involved in the resistance to TRAIL, we examined whether overexpression of DR5 would induce sensitivity to rTRAIL in DLD-1/TRAIL and MCF10A cells, both of which have characteristics of TRAIL-resistance. The expression level of DR5 in these cells after transfection with a DR5 expression vector was estimated by Western blot analysis (Fig. 9A and B). Then, we examined the anti-proliferative effect of rTRAIL at various concentrations on the transfected cells as judged from the cell viability assessed by the using trypan blue dye-exclusion test (Fig. 9A and 9B). The cells transfected with the control or DR5 expression vector were treated with rTRAIL for 24 h. Overexpression of DR5 resulted in significant growth suppression on rTRAIL-treated cells compared with the suppression found for the cells transfected with the control vector. The IC_50_ value of rTRAIL for each cell line shifted to a slightly lower concentration compared with that before the transfection. However, the level of apoptotic response to TRAIL was not fully recovered to become similar to that for each TRAIL-sensitive cell line. Also, the activation of caspase-8 and caspase-3 were significantly increased in the cells transfected with the DR5 expression vector in MCF10A cells, but not in the case of the DLD-1/TRAIL cells (Fig. 9C and 9D). We concluded that the overexpression of DR5 did not improve the impaired recruitment of DR5 to cell surface membrane significantly. These results indicated that the main mechanism of TRAIL-resistance was malfunction of its recruitment on the cell surface.

### Growth inhibition of 3-D tumour spheroids by combined treatment with α-mangostin and rTRAIL

**Figure 10:**
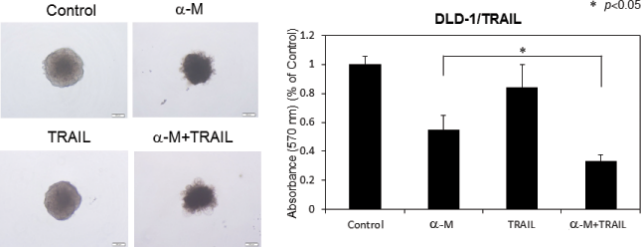
α-Mangostin and rTRAIL induced significant growth suppression of 3-D tumor spheroids TRAIL-resistant DLD-1 cells under conditions for spheroid formations were treated with α-mangostin (30 μM) and/or rTRAIL (100 ng/ml) for 24 h. The absorbance at 570 nm was measured for each well, and the average and standard deviations were calculated for each sample. The control was DMSO alone.

In order to further estimate the growth inhibitory effect of α-mangostin, we examined the synergistic anti-proliferative effects of α-mangostin (30 μM) and rTRAIL (100 ng/ml), on DLD-1/TRAIL cells cultured as 3-D spheroids, instead of as evaluated by the *in vivo* assay (Fig. 10). As a result, the combination treatment with α-mangostin and rTRAIL resulted in synergistic tumour growth suppression of these cells. The formation of tumour spheroids by the cells treated with α-mangostin and rTRAIL was significantly suppressed from the surrounding compared with the formation by the DLD-1/TRAIL cells treated with rTRAIL alone. These results indicated that α-mangostin induced TRAIL-sensitivity even in 3-D tumour spheroids as well as *in vitro*.

## DISCUSSION

**Figure 11:**
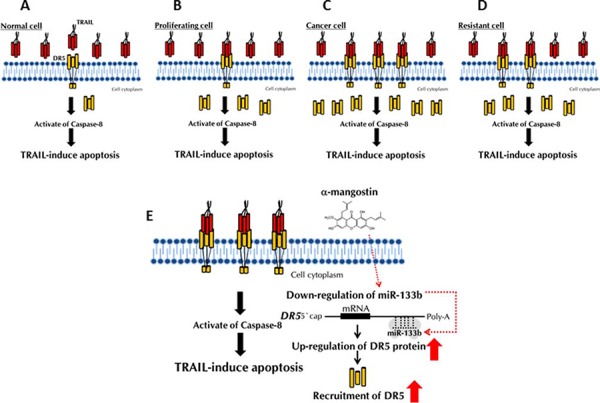
Schematic diagram of the TRAIL-resistance mechanism and machinery involved in the TRAIL-induced apoptosis by α-mangostin **A, B, C** and **D.** The mechanism of TRAIL-resistance consisted of not only a decrease in the expression level of DR5 and also malfunction of its recruitment to the cell surface. **E.** The effects of a-mangostin are indicated by red arrows.

The current study aimed at developing an adjuvant compound for cytokine therapy to conquer TRAIL-resistance, because TRAIL-receptors are expressed on the tumour cell surface, without affecting normal cells. The detailed mechanisms of TRAIL-resistance are not well understood. So far, many studies have reported that the main mechanism is down-regulation of DR5, which is common to various cancer cell types [11, 16]. In this study, we clarified that the mechanism of TRAIL-resistance consisted of not only a decrease in the expression level of DR5 and also malfunction of its recruitment to the cell surface, as evidenced form the results obtained by using TRAIL-resistant human colon cancer DLD-1 cells and human mammary gland epithelial MCF10A cells and their cancer stem-like cells (Fig. 1C). The malfunction of DR5 recruitment was also observed in human mammary gland epithelial MCF10A cells, which have characteristics of TRAIL-resistance (Fig. 6). When these TRAIL-resistant cell lines were made to exogenously overexpress DR5, sensitivity to TRAIL was not fully recovered (Fig. 9A and 9B), which indicated that both mechanisms co-existed to account for the resistance. Based on these results, we propose that the TRAIL-receptor DR4/5 was present, but at an extremely low amount and also that the impaired oligomerization could exist in normal cells (Fig. 11A). Therefore, TRAIL would hardly bind to normal cells. In proliferating non-cancerous cells such as MCF10A cells (Fig. 11B), the amounts of the receptor molecules would be more than those on normal cells, but TRAIL-binding would not be frequent compared with that by cancer cells (Fig. 11C). In cancerous cells, the amount of receptor molecules would be abundant and oligomerization efficiently performed in response to TRAIL exposure, leading to frequent binding of TRAIL to its receptors (Fig. 11C). In DLD-1/TRAIL cells, decreased expression of the receptors and dysfunction of oligomerization would co-exist, leading to resistance to TRAIL-induced apoptosis (Fig. 11D). Our data indicate that the main mechanism of TRAIL-resistance was malfunction of its recruitment to the cell surface membrane. On the other hand, we did not find any malfunction of the caspase-8 activation machinery. Notably, α-mangostin cancelled the 2 resistance machineries by increasing the expression level of DR5 through down-regulation of miR-133b and efficient recruitment of DR5 in DLD-1 and their TRAIL-resistant cells (Figs. 2, 3, and 5B), but not in the non-cancerous TRAIL-resistant MCF10A cells. These findings strongly indicated that α-mangostin could contribute to chemoprevention of cancer development by cooperating with TRAIL.

We demonstrated that the level of intracellular miR-133b was down-regulated after the treatment with α-mangostin (Fig. 5B); whereas DR5, a target gene of miR-133b, was clearly up-regulated (Figs. 2 and 5A). The 3- to 4-fold decrease in the miR-133b level by α-mangostin actually up-regulated DR5 (Fig. 5B). Also, the gene silencing of DR5 suppressed apoptosis significantly, which would have considerably contributed to the TRAIL-induced apoptosis by α-mangostin (Fig. 4), indicating that the apoptosis was at least in part due to the up-regulation of DR5 by the down-regulation of miR-133b. Hagiwara *et al*. reported that stilbene derivatives promote the expression of Argonaute2 (Ago2), resulting in the elevated expression of certain miRNAs [17]. Therefore, it is also possible that such natural compounds may have affected some miRNA expression by modulating RNase III family members Drosha and Dicer. Additionally, α-mangostin effectively induced the transfer of DR5 to the cancer cell surface membrane and improved the DR5 oligomerization. It has been reported that the down-regulation of surface expression of DR5 may be attributed to the co-localization with LC3-II in the autophagosomes of TRAIL-resistant human breast cancer cell lines [10]. When we analyzed the steady-state expression level of LC3-II, however, no difference was observed between TRAIL-sensitive and -resistant DLD-1 cell lines (Supplementary Fig. 2). Thomas *et al*. reported that doxorubicin induces the translocation of DR5 from the cytoplasm to the tumour cell surface [7]. The mechanism of this translocation has not yet been established, and there is concern about possible side effects. In 2003, it was reported that activated Cdc-42-associated kinase (Ack1) is required for DR4 recruitment to lipid rafts and for induction of TRAIL-induced cell death [18]. We checked the steady-state expression level of Ack1 between DLD-1 and DLD-1/TRAIL cell lines; however, the expression level was similar in each cell line (Supplementary Fig. 3). In addition, α-mangostin did not affect the Ack1 level either cell line. Thus, the mechanism of DR5 oligomerization is not yet clearly understood. Based on the results of this study, we emphasize the point that α-mangostin was a useful compound against TRAIL-resistant cells as well as cancer-stem like cells (Figs. 7 and 8). Cancer stem cells are highly resistant to anti-cancer drugs, because these cells show slow growth and are recruited into the G_0_-phase of the cell cycle. Our study is the first to show that cancer stem-like cells, which expressed the CSC markers CD44 and ABCG2 as well as the stem-cell marker SOX2 [19], exhibited significant sensitivity to TRAIL-induced apoptosis and that α-mangostin functioned as a sensitizer of TRAIL-induced apoptosis by inducing recruitment of DR5 to the cancer cell surface (Fig. 8) [15]. Therefore, these results indicated that α-mangostin would be a useful compound for immune-cytokine therapy. Recently, clinical trials using combination treatment with anti-DR5 antibody and chemotherapeutics have begun; however, hepatic dysfunction and various adverse effects are a serious problem [20]. Since chemotherapeutic drugs lead to DNA damage in cells, combination treatment using TRAIL with these cytotoxic agents, even at lower doses than those used in conventional chemotherapy, can result in severe side effects. Importantly, α-mangostin, which is a naturally-occurring compound can prevent these problems caused by chemotherapeutics.

In conclusion, we newly found that the mechanism of TRAIL-resistance consisted of a decrease in the expression level of DR5 and malfunction of its recruitment to the cell surface. α-Mangostin, a xanthone derivative, cancelled this resistance by increasing the expression level of DR5 through down-regulation of miR-133b and effectively inducing the transfer of DR5 from the cytoplasm to the tumour cell surface membrane (Fig. 11E). Further studies will be needed to elucidate how α-mangostin induced DR5 oligomerization at the tumour cell surface membrane. Our findings strongly indicated that α-mangostin functioned as a sensitizer of TRAIL-induced apoptosis and could become a possible adjuvant compound for cytokine therapy to conquer TRAIL-resistance in TRAIL-resistant cancer cells and in cancer-stem cells.

## MATERIALS AND METHODS

### Cell culture and cell viability

Human colon cancer cell line DLD-1 and TRAIL-resistant DLD-1 cell line, the latter of which was obtained from DLD-1 cells after selection by drug pressure, were grown in RPMI-1640 medium supplemented with 5% (v/v) heat-inactivated FBS and 2 mM L-glutamine under an atmosphere of 95% air and 5% CO_2_ at 37°C. Human cancer stem-like cell lines, iCSCL-10A1 and iCSCL-10A2 were established from mammary gland epithelial cell line MCF10A as previously reported [15]. In this paper, these cancer stem-like cell lines were named as abbreviations, CSC-1 and CSC-2, respectively. MCF10A cells were grown in MEBM medium (Lonza, Tokyo, Japan); and CSC-1 and CSC-2 cells, in DMEM medium (Invitrogen, San Diego, CA, USA). The number of viable cells was determined by performing the trypan blue dye-exclusion test. TRAIL was obtained from BioVision (Milpitas, California, USA). In some experiments the cells were co-incubated with DMSO or PBS alone as a control.

### Quantitative reverse-transcriptase PCR using real-time PCR

Total RNA was extracted from cells by TRIzol containing phenol/guanidium isothiocyanate (Invitrogen) and then treated with DNase I. The RNA was subsequently reverse-transcribed to cDNA by using Super-Script III reverse transcriptase (Invitrogen) according to the manufacturer's protocol. In order to examine the expression level of mature miR-133b in detail, we performed TaqMan^®^ MicroRNA Assays (Applied Biosystems, Foster City, CA) using real-time PCR [21]. The threshold cycle (Ct) is defined as the fractional cycle number at which the fluorescence passes a fixed threshold. The expression level of the miRNA in each sample was measured and was normalized to *miR-21* expression, which was used as an internal control. Calculation of the Ct value was done by using the second-derivative maximum method, and relative quantification was made by the comparative Ct method. All reactions were run in triplicate. The PCR primer pairs for miR-133b and -21 were obtained commercially from Applied Biosystems.

### Western blotting

Cells were homogenized in chilled lysis buffer comprising 10 mM Tris-HCl (pH 7.4), containing 1% NP-40, 0.1% deoxycholic acid, 0.1% SDS, 150 mM NaCl, 1 mM EDTA, and 1% Protease Inhibitor Cocktail (Sigma, Tokyo, Japan) and stood for 20 min on ice. After centrifugation at 13, 000 rpm for 20 min at 4°C, the supernatants were collected as protein samples. Protein contents were measured with a DC Protein assay kit (Biorad, Hercules, CA). Ten micrograms of lysate protein for Western blotting was separated by SDS-PAGE using a 7.5% or 10% polyacrylamide gel and electroblotted onto a PVDF membrane (Amersham Biosciences, Piscataway, NJ). After blockage of nonspecific binding sites for 1 h with 5% nonfat milk in PBS containing 0.1% Tween 20, the membrane was incubated overnight at 4°C with various primary antibodies. They included anti-PARP-1(Santa Cruz Biotechnology, Santa Cruz, CA), anti-human FADD (MBL, Nagoya, Japan), anti-human cleaved caspase-8 (Cell Signaling Technology Inc.), and anti-human Δ-actin antibodies (Sigma). The membranes were then washed 3 times with PBS containing 0.1% Tween 20, incubated further with HRP-conjugated sheep anti-mouse or donkey anti-rabbit IgG antibody (Cell Signaling Technology Inc.) at room temperature, and then washed 3 times with PBS containing 0.1% Tween 20. The immunoblots were visualized by use of an enhanced chemiluminescence detection kit (PerkinElmer, Inc., Waltham, MA, USA).

### Immunocytochemistry

DLD-1 and DLD-1/TRAIL cells were seeded into the wells of a Lab-Tek II Chamber Slide System (Thermo Fisher Scientific Inc., Waltham, MA), each well containing 1.0 ml of culture medium plus 10% (w/v) fetal bovine serum. After 48 h of treatment, the cells were immunostained with anti-DR5 antibody according to the immunofluorescence protocol of Cell Signaling Technology. The nuclei were stained with Hoechet33342, and for actin labeling the cells were incubated with the fluorescent F-actin probe Rhodamine Phalloidin (Cytoskeleton, Denver, CO). The cells were observed with a BIOREVO fluorescence microscope (Keyence, Osaka, Japan).

### Cell transfection with miRNA or siRNA

DLD-1 and DLD-1/TRAIL cells were seeded into 6-well plates at a concentration of 0.5–1.0 × 10^5^/1ml/well on the day before the transfection. The siRNA for *DR5* (siR-DR5: 2 nM) was used for the transfection of the cells, which was achieved by using cationic liposomes, i.e., Lipofectamine RNAiMAX (Invitrogen), according to the manufacturer's lipofection protocol. The sequence of miR-133b was 5′-UUUGGUCCCCUUCAACCAGCUA-3′; and that of siRNA for *DR5*, 5′-GAAGACGGTAGAGAT TGCATCTCCT-3′ (siR-DR5). We used non-specific control (NC) Duplex VII (57% GC Content; Dharmacon Research, Inc., Lafayette, CO, USA) as a control. The effects manifested by the introduction of the mature miRNA or siRNA into the cells were assayed at 48 or 72 h, respectively, after the transfection.

### Assay for iuciferase activity

We constructed sensor vectors by joining the region with or without a possible binding site from the 3′-UTR of human *DR5* (No.4271–4650) with a luciferase reporter pMIR-control vector (Ambion, Foster City, CA, USA) to examine the target sequence of miR-133b. To generate sensor vectors with 4 mutations in the binding site of the 3′-UTR of human *DR5* (No.4455–4463) for miR-133b, we mutated seed regions from GGACCAAA to GGCATGAA (mt-*DR5*, PrimeSTAR^®^ Mutagenesis Basal Kit; TaKaRa). The sensor vector with these mutations was submitted to Life Science Research Center, Gifu University, for DNA sequencing. The cells were seeded in 12-well plates at a concentration of 0.5 × 10^5^/well the day before the transfection. The sensor vector (concentration; 0.5 μg/well) and 10 nM miR-133b or nonspecific control miRNA (Dharmacon) were used for the co-transfection of the cells by using Lipofectamine RNAiMAX (Invitrogen). Forty-eight hours after the co-transfection, luciferase activities were measured by using a Dual-Glo™ Luciferase Assay System (Promega, Madison, WI, USA) according to the manufacturer's protocol. Firefly luciferase activity was normalized to *Renilla* luciferase activity.

### Assay for DR5 overexpression

The DR5 expression vector was generated by inserting the open reading frame of DR5 cDNA into the SgfI and PmeI site of the pF5A-CMV *neo* vector (Promega). DLD-1/TRAIL and MCF10A cells were seeded into 6-well plates at a concentration of 0.5 × 10^5^/well and transfected at 0.4 μg/well with the control vector or pF5A-DR5 expression vector by using Lipofectamine 2000 (Invitrogen) at 24 h after the transfection with rTRAIL. The effects manifested by DR5 overexpression were assayed at 24 h after the transfection with plasmids.

### 3-D spheroid colorimetric viability assay

DLD-1/TRAIL cells were seeded into a 96-well plate at 3000 cells/well. Dispense 50 μl of single cell suspension in 1 × Spheroid Formation ECM (Trevigen) per well was dispensed into a 3D culture spheroid formation plate. The plate was then centrifuged at 200 × g for 3 minutes at room temperature, after which it was incubated at 37°C in a tissue culture incubator for 48 hours to promote spheroid formation. After 48 h, 50 μl of warm cell culture medium containing the desired compound was added; and the plate was then incubated at 37°C in the tissue culture incubator for 2 days. After 48 h, a one-tenth volume (10 μl/100 μl) of MTT reagent was added to each well; and then the plate was transferred back to incubator at 37°C for 24 hours. After 24 hours, an equal volume (100 μl/100 μl) of warm detergent reagent at 37°C was added to each well; and then the plate was placed back in the incubator for 24 h to solubilize the cells and to release the MTT crystals formed. The absorbance at 570 nm was then read.

### Statistics

Differences were statistically evaluated by one-way ANOVA followed by the *t*-test. A *p*-value of less than 0.05 was considered to be statistically significant.

## SUPPLEMENTARY FIGURES


