# Supplementary material for: Understanding of tolerance in TRAIL-induced apoptosis and cancelation of its machinery by α-mangostin, a xanthone derivative
Source: Oncotarget. 2015 Jul 16;6(28):25828–42. doi: 10.18632/oncotarget.4558 (PMC4694869; doi:10.18632/oncotarget.4558)
Supplement: Supplementary file 1 [file oncotarget-06-25828-s001.pdf]

SUPPLEMENTARY FIGURES

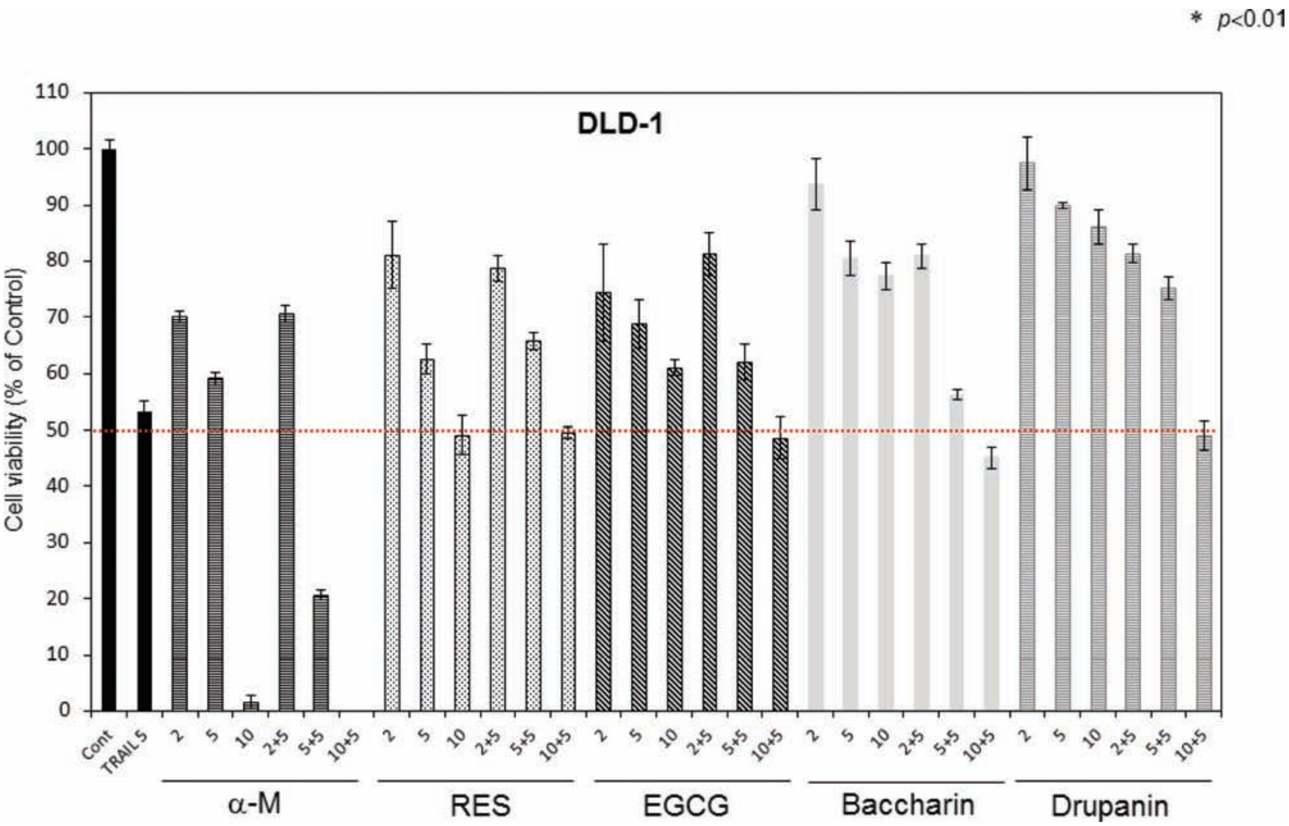

Supplementary Figure S1: DLD-1 cells were treated with α-mangostin, resveratrol, epigallocatechin-3-gallate, baccharin and drupanin (2, 5, 10 µM) and/or rTRAIL (5 ng/ml) for 48 h. Data were obtained from 3 independent experiments. The cell viability of the control (0; DMSO alone) is indicated as 100%.

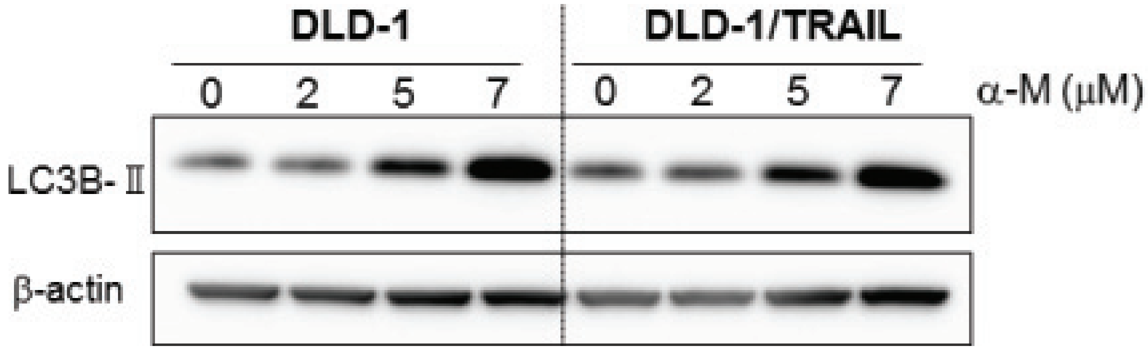

Supplementary Figure S2: TRAIL-sensitive DLD-1 and -resistant DLD-1 cells were treated with α-mangostin (2, 5, 7 μM) for 48 h. Western blot analysis was performed to determine the expression of LC3B-II, with β-actin as an internal control.

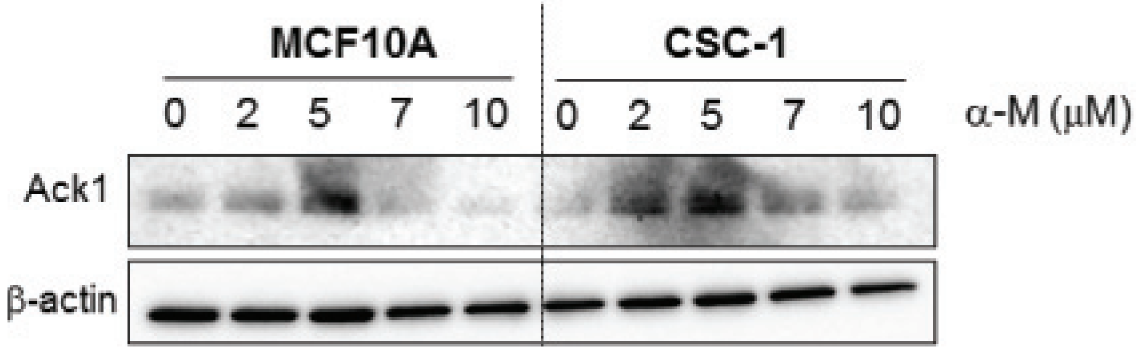

Supplementary Figure S3: TRAIL-sensitive DLD-1 and -resistant DLD-1 cells were treated with α-mangostin (2, 5, 7, 10 μM) for 48 h. Western blot analysis was performed to determine the expression of Ack1. β-Actin was used as the internal control.
